# Antiatherosclerosis Properties of Total Saponins of Garlic in Rats

**DOI:** 10.1155/2020/3683659

**Published:** 2020-02-27

**Authors:** Qing Miao, Ruihai Wang, Dong Bai, Xin Xue, Jing Xu, Xiaoxin Sun, Limei Liu

**Affiliations:** Institute of Basic Theory for Chinese Medicine, China Academy of Chinese Medical Sciences, Beijing 100700, China

## Abstract

Garlic has been proven effective in the prevention and treatment of atherosclerosis (AS), which is widely used as a food and medicine by people in daily life. Garlic saponins are the main active nonsulfur compounds of garlic, which have a variety of pharmacological activities against cardiovascular diseases. In this study, the antiatherosclerosis properties and mechanism of total saponins of garlic (TSG) in rats were explored. The AS animal model was established by a combination of high-fat feeding, intraperitoneal injection of vitamin D_3,_ and ovalbumin-induced inflammation in SD rats. Then, the atherosclerotic rats were gavaged daily by TSG for 4 weeks. Administration of TSG markedly decreased atherosclerotic lesions in the aorta of atherosclerotic rats. TSG restored the serum lipid profile by significantly decreasing the lipid levels and had effective antioxidation by inhibiting the content of malondialdehyde (MDA) and restoring the reduced activity of superoxide dismutase (SOD). Additionally, the ratio of thromboxane B_2_ (TXB_2_) and 6-keto-prostaglandin F_1*α*_ (6-keto-PGF_1*α*_) could be maintained in a relatively stable dynamic balance after administration of TSG to maintain the vascular homeostasis. In summary, TSG had therapeutic effects on AS, which are promising as functional foods or nutraceuticals for the prevention and treatment of AS.

## 1. Introduction

Atherosclerosis (AS) is one of the leading risk factors of cardiovascular diseases. With the improvement of living standards and the changes in people's dietary habits, the cardiovascular diseases caused by AS are increasing [1]. AS has become the principal causes of morbidity and mortality worldwide. Increasing evidence supporting diet plays a crucial role in the prevention and treatment of AS [2]. Meanwhile, with the spread of complementary and alternative medicine, the functional food has been accepted because of its diversity, easy access, safety, and relatively low cost [3]. Therefore, it is essential to investigate the functional food with antiatherosclerosis.

Garlic (*Allium sativum* L.) is commonly consumed as a flavor and a medicine since antiquity and now cultivated all over the world [4]. As a medicine and food homologous traditional Chinese medicine, garlic is widely used by people in their daily life in China [5]. Substantial pharmacological studies have shown garlic has a variety of biological activities [6–14], including antioxidant, antibacterial, antifungal, anti-inflammatory, and antiobesity activities, which can effectively prevent the occurrence and development of AS. These functions are mainly due to its diverse bioactive compounds. There are two major categories of active compounds that are currently under concern: one is fat-soluble compounds, mainly garlic oil, and the other is water-soluble compounds, mainly saponins and polysaccharides. In recent years, investigations have increasingly focused on garlic oil, and various products made from garlic oil have been developed. But, because of the strong irritancy and instability, the development and application of garlic oil has been limited. Garlic saponins are the main active nonsulfur compounds of garlic [15]. It is proved that garlic saponins are more stable in the cooking process [16] and have various beneficial pharmacological activities, such as antifungal, antitumor, antithrombotic, and cholesterol-lowering effects [17]. Studies have confirmed that garlic saponins have preventive effects on the formation of thrombus, which can significantly inhibit platelet aggregation and prolong blood clotting time [18]. Thus, it can hypothesize that garlic saponin may have potential effects on the treatment of AS and may be a promising complementary and alternative medicine for atherosclerosis.

In this study, the total saponins of garlic (TSG) were extracted from *Allium sativum* L. to evaluate the effects on AS and explore the underlying mechanisms. The AS model rats were established by a combination of high-fat feeding, intraperitoneal injection of vitamin D_3,_ and ovalbumin-induced inflammation. The antiatherosclerosis property of TSG was verified by assaying the effects on the aortic plaque, blood lipids, antioxidant factors, and vascular homeostasis of rats.

## 2. Materials and Methods

### 2.1. Preparation of TSG

Garlic was purchased from an authentic local market (Beijing, China). The batch of garlic was from the garlic planting base and identified by Jinxiang Agricultural Bureau (Jining, China). The extraction method of TSG was based on our previous study [19–21]. After extracting garlic volatile oil by steam distillation, the double extraction method was used to extract the total polysaccharide with water and extract the total saponin with alcohol, respectively. After purification by macroporous resin, the content of total saponins in garlic extract was more than 65%.

### 2.2. Animals and Drug Administration

All experimental procedures were approved by the Laboratory Animal Ethics Committee of the Institute of Basic Theory of Chinese Medicine, China Academy of Chinese Medical Sciences (ethical approval number: 2011-049). Male Sprague Dawley rats (200 ± 20 g of body weight) were provided by National Institutes for Food and Drug Control (license ^#^SCXK 2009-0017, Beijing, China). The rats were adapted to an environmentally controlled breeding room and fed with food and water *ad libitum*.

After an adaptation period of a week, the rats were randomly divided into 2 groups, namely, the control group (*n* = 15) and experimental group (*n* = 70). The rats in the control group were given a normal diet and intraperitoneally injected with 0.9% saline solution. The rats in the experimental group were fed a high-fat diet and intraperitoneally injected with vitamin D_3_ (600000 IU/kg, once). Meanwhile, these rats were subcutaneously injected with an antigen emulsion containing ovalbumin (3 mg/kg) at multiple points in the back. After 3 weeks, the immune response of rats was stimulated by intraperitoneal injection with ovalbumin (2.5 mg/kg, once a week). After 8 weeks, 3 rats in the control group and 10 rats in the experimental group were randomly selected, and their aortas were stripped and stained with hematoxylin-eosin (HE) reagents to confirm the formation of AS. Afterwards, the atherosclerotic rats were assigned randomly to 5 groups (*n* = 12 in each group): the model group, positive control group (0.1 mg/kg simvastatin), low-dose group (0.6 g/kg TSG), medium-dose group (1.2 g/kg TSG), and high-dose group (2.4 g/kg TSG). Rats in treatment groups were intragastrically administrated with simvastatin or TSG for 4 weeks. Rats in control and model groups were intragastrically administrated with the same volume of 0.9% saline solution for 4 weeks. After the last administration, all rats were sacrificed after being anesthetized by intraperitoneal injection with pentobarbital. Blood samples were taken from the abdominal aorta, and the aortas were rapidly stripped from the aortic root to the iliac branch for further analyses.

### 2.3. Histopathology

After thoroughly removing adventitial fat and connective tissue, the aortas were fixed in 4% paraformaldehyde and dehydrated in graded ethanol and embedded in paraffin. Paraffin sections were sliced using a slide microtome and dewaxed using xylene. Then, these sections were stained with HE reagents for observation. Photographs were blindly taken at random fields under an optical microscope (Olympus Co., Ltd., Tokyo, Japan).

### 2.4. Serum Analyses

Blood samples were centrifuged at 3000 rpm for 15 minutes in 4°C, and the serum was collected for later detection. Serum levels of total cholesterol (TC), total triglyceride (TG), low-density lipoprotein cholesterol (LDL-C), and high-density lipoprotein cholesterol (HDL-C) were measured by using kits (Beijing North Kangtai Clinical Reagent Co. Ltd., Beijing, China) according to the manufacturer's instructions. Levels of malondialdehyde (MDA) and superoxide dismutase (SOD) in serum were measured using a GF-D800 semiautomatic biochemistry analyzer (Shandong Gaomi Caihong Analysis Instrument Co., Ltd., Gaomi, China). Levels of thromboxane B_2_ (TXB_2_) and 6-keto-prostaglandin F_1*α*_ (6-keto-PGF_1*α*_) in serum were measured by radioimmunoassay.

### 2.5. Statistical Analyses

Data were presented as mean ± SD, and the results were analyzed using SPSS 17.0 software. The significance of difference was determined by one-way ANOVA. Values of *p* < 0.05 were considered statistically significant.

## 3. Results

### 3.1. Histopathological Analysis

**Figure 1:**
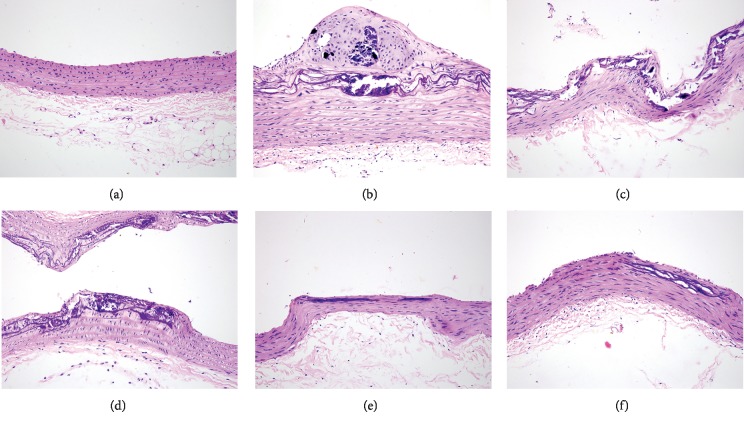
Effects of different treatment on histopathological changes in the aortas of rats: (a) the control group, (b) model group, (c) positive control group, (d) 0.6 g/Kg TSG group, (e) 1.2 g/Kg TSG group, and (f) 2.4 g/Kg TSG group.

The AS model rats were established by a combination of high-fat feeding, intraperitoneal injection of vitamin D3, and ovalbumin-induced inflammation. After 8 weeks, the aortas of the selected rats were stripped for pathological observation. Compared with the normal rats, obvious atherosclerotic lesions could be observed in the AS model rats (Figures 1(a) and 1(b)). Prominent lipid plaque beneath ascular intima was found, and tissue necrosis, cholesterol crystallization, and calcification were seen in the plaques, indicating the AS model was successfully established. Subsequently, the atherosclerotic rats were treated with simvastatin and different doses of TSG for 4 weeks. Histopathological examinations exhibited the atherosclerotic lesions of rats in each group were improved to different extents (Figures 1(c)–1(f)). After administration, the histology of the aortas was significantly improved, and the lipid deposition, cholesterol crystallization, and calcification were reduced, especially the rats in 1.2 g/Kg and 2.4 g/Kg TSG groups, indicating TSG could attenuate the atherosclerotic lesion of the rats.

### 3.2. Effects on Serum Lipid Levels in Rats

After the experiment, the serum TC, TG, and LDL-C levels of the rats in the model group were found to be significantly higher than those in the control group. Compared with the model group, TC and TG levels of the rats were significantly decreased after treating with simvastatin and different doses of TSG, respectively. LDL-C levels of the rats were also decreased after administration, especially the rats treated simvastatin, 1.2 g/Kg and 2.4 g/Kg TSG. However, there was no significant difference in HDL-C level between groups. The results indicated that TSG decreased the serum lipid levels dose-dependently. The specific data are shown in Table 1.

### 3.3. Effects on Levels of MDA and SOD in Rats

**Figure 2:**
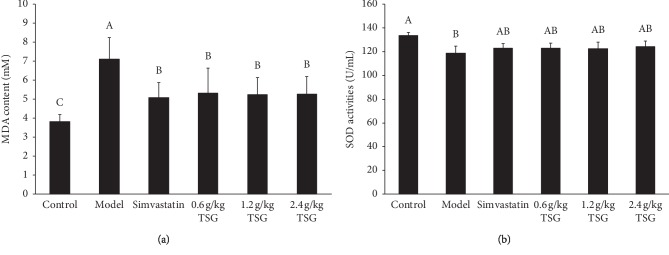
Effects of TSG on MDA (a) and SOD (b) levels in rats. Data are presented as mean ± SD, (*n* = 12). Values with different capital letters in a column are significantly different (*p* < 0.05) from each other.

The effects of TSG on the levels of MDA and SOD were investigated, and the results are shown in Figure 2. Compared with the control group, MDA contents of the rats in the model group were significantly increased, and SOD activities were significantly decreased, demonstrating the induction of AS was associated with oxidative stress. After administration of simvastatin and TSG, MDA contents of rats were significantly decreased, and SOD activities were slightly increased without statistically significant differences.

### 3.4. Effects on Serum Levels of TXB2 and 6-Keto-PGF1*α* in Rats

**Figure 3:**
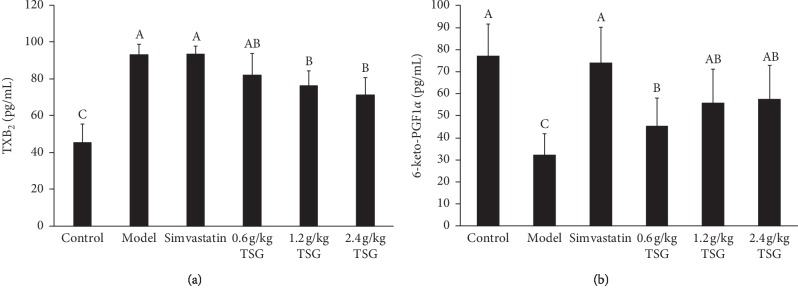
Effects of TSG on TXB_2_ (a) and 6-keto-PGF_1*α*_ (b) levels in rats. Data are presented as mean ± SD, (*n* = 12). Values with different capital letters in a column are significantly different (*p* < 0.05) from each other.

The effects of TSG on the serum levels of TXB_2_ and 6-keto-PGF_1α_ were investigated, and the results are shown in Figure 3. Compared with the control group, the TXB_2_ levels of the rats in the model group were significantly increased, and the 6-keto-PGF_1*α*_ levels were significantly increased. Treated with simvastatin and TSG, the levels of TXB_2_ were significantly decreased, and the 6-keto-PGF_1*α*_ levels were significantly increased, especially in the rats treated with 1.2 g/Kg and 2.4 g/Kg TSG. Additionally, the ratio of TXB_2_ and 6-keto-PGF_1*α*_ in 0.6 g/Kg, 1.2 g/Kg, and 2.4 g/Kg TSG groups were less than that in the model group by 37.35%, 52.83%, and 57.06%, respectively.

## 4. Discussion

Saponins, composed of sapogenin and sugar chain, are the important active compounds of plants. In recent decades, the research on saponins has been extended to extraction, isolation, structural identification, and pharmacological activities of pure compound and total saponins [22–24]. It is found that saponins had the effects of antioxidation, antiplatelet aggregation, vasodilation, and lowering blood lipids, which provide a potential new natural source of the prevention and treatment of cardiovascular diseases [25].

Garlic has been proven effective in the prevention and treatment of AS [26]. However, as the main active nonsulfur compounds of garlic, the antiatherosclerotic effects of TSG are still unclear. In the present study, we investigated the effects of TSG on atherosclerotic rats. Considering plaque formation is a crucial characteristic of the progression of atherosclerotic disease [27], we first examined the pathological change in the aorta and evaluated the inhibitory effects of TSG on the atherosclerotic lesion in rats. Histopathological examinations revealed obvious lipid plaque beneath vascular intima in atherosclerotic rats. Administration of TSG markedly improved the pathological change of the aortas in atherosclerotic rats, indicating TSG have a certain therapeutic effect on AS.

Dyslipidemia and oxidative stress are the major pathological factors in the occurrence and development of AS [28, 29]. The current studies have found that the saponin fractions from garlic can reduce plasma cholesterol levels in hyperlipidemia rats and that total saponins are the primary antihypoxia active compounds of garlic [30–32]. In this study, we found that TSG corrected the lipid-related parameters to significantly decrease the lipid levels of atherosclerotic rats, which manifested TSG could restore the serum lipid profile of rats with AS. In addition, TSG showed effective antioxidation by inhibiting the content of MDA and restoring the reduced activity of SOD. The results indicated that TSG could prevent AS through lowering blood lipids and antioxidation. These results are consistent with the previous research findings.

TXA_2_ and PGI_2_ are metabolites of arachidonic acid and important vasoactive substances that stimulate the homeostasis of the cardiovascular system [33]. TXA_2_ and PGI_2_ have the opposite effects *in vivo* [34]. TXA_2_ induces vasoconstriction and platelet aggregation and promotes AS formation [35], whereas PGI_2_ has the function of relaxing blood vessels and inhibiting platelet aggregation [36]. The balance between TXA_2_ and PGI_2_ maintains the basic function of blood vessels and platelets, ensuring the stable blood circulation, which plays a vital role in the prevention and treatment of AS [37]. Since TXA_2_ and PGI_2_ are both unstable *in vivo*, it is difficult to measure them directly. The levels of TXA_2_ and PGI_2_ are usually determined by measuring their stable metabolites TXB_2_ and 6-keto-PGF_1*α*_, respectively. In this study, we found that TSG could lower the level of TXB_2_ and elevate the level of 6-keto-PGF_1*α*_. After administration of TSG, the ratio of TXB_2_ and 6-keto-PGF_1*α*_ could be maintained in a relatively stable dynamic balance to maintain the vascular homeostasis.

## 5. Conclusions

Taken together, we provided the evidences that TSG had therapeutic effects on atherosclerotic rats by lowering blood lipids, inhibiting oxidation, and maintaining vascular homeostasis. These discoveries suggested that TSG might mainly contribute to the antiatherosclerosis effect of garlic, which could be extended to investigate the antiatherosclerotic effects of plants enriched with saponins. Consequently, garlic saponins are promising as functional foods or nutraceuticals for the prevention and treatment of AS. The effects of garlic and its saponins on cardiovascular diseases remain to be further validated by animal experiments and clinical trials, and the underlying mechanisms need to be further investigated.

## Figures and Tables

**Table 1:** Effects of TSG on serum lipid levels in rats.

Group	TC (mM)	TG (mM)	HDL-C (mM)	LDL-C (mM)
Control	1.684 ± 0.211 C	0.087 ± 0.012 AB	1.332 ± 0.220	0.181 ± 0.073 C
Model	4.542 ± 0.987 A	0.108 ± 0.025 A	1.553 ± 0.380	0.770 ± 0.322 A
Simvastatin	2.950 ± 0.584 B	0.069 ± 0.018 B	1.709 ± 0.290	0.359 ± 0.162 B
0.6 g/kg TSG	3.463 ± 0.633 B	0.083 ± 0.016 AB	1.599 ± 0.344	0.470 ± 0.323 AB
1.2 g/kg TSG	3.164 ± 0.755 B	0.084 ± 0.023 AB	1.557 ± 0.260	0.334 ± 0.196 B
2.4 g/kg TSG	3.143 ± 0.776 B	0.074 ± 0.019 B	1.468 ± 0.196	0.225 ± 0.138 B

Data are presented as mean ± SD, (*n* = 12). Values with different capital letters in a column are significantly different (*p* < 0.05) from each other.

## Data Availability

The data used to support the findings of this study are included within the article.
